# Chest computed tomography findings in more than 4,000 non-hospitalized suspected COVID-19 patients

**DOI:** 10.22088/cjim.13.0.187

**Published:** 2022

**Authors:** Naser Ghaemian, Reza Ghadimi, Soraya Soraya, Simin Mouodi

**Affiliations:** 1 Cancer Research Center, Babol University of Medical Sciences, Babol, Iran; 2 Social Determinants of Health Research Center, Health Research Institute, Babol University of Medical Sciences, Babol, Iran; 3 Department of Epidemiology and Biostatistics, School of Public Health, Babol University of Medical Sciences, Babol, Iran

**Keywords:** Tomography, Coronavirus, Diagnosis

## Abstract

**Background:**

When the first wave of COVID-19 outbreak occurred, the infrastructure for definitive detection of the disease through real-time polymerase chain reaction (RT-PCR) was not yet available in many regions, and a large proportion of suspected patients were inevitably referred to radiology centers to provide a chest CT scan. This research was conducted to describe chest CT characteristics in patients who underwent chest CT during the first weeks of COVID-19 outbreak in Babol, Iran.

**Methods:**

All non-hospitalized suspected COVID-19 patients referred to the state radiologic clinic to perform chest CT from March 8, 2020 to March 28, 2020 have been enrolled in this observational study. All CT scans were reviewed by a faculty member radiologist with approximately 20 years of experience.

**Results:**

Totally, 2,207 (52.3%) men and 2016 (47.7%) women have been examined. Imaging characteristics in 2292 (54.3%) individuals illustrated a highly suggestive sign of COVID-19 infection while 1869 (44.3%) had a normal chest CT scan. 1813 cases (77.00%) had bilateral involvement and 541 cases (23.00%) were infected unilaterally; Also, 1727 (73.36%) patients had left-sided involvement. Lung field involvement in 2036 (86.49%) patients was less than 20%. Ground glass opacity had a sensitivity, specificity, PPV, NPV, LR+ and LR- of 99%, 96%, 96%, 98%, 22 and 0.01, respectively, for categorization of a patient as a COVID-19 case. These values were 99%, 73%, 70%, 99%, 3.72% and 0.01%, respectively for consolidations.

**Conclusion:**

Although, RT-PCR is still introduced as the gold standard method for definite diagnosis, diagnostic accuracy of chest CT in COVID-19 detection is considerable.

The probability of respiratory involvement in patients with coronavirus disease 2019 (COVID-19) has led the physicians to refer suspected patients to radiologic centers to provide a chest computed tomography (CT) (1, 2). Fever, cough, fatigue, increased sputum production, shortness of breath and myalgia have been listed as the most common clinical symptoms in patients with COVID-19 (2). World Health Organization (WHO) has provided interim guidelines to implement proper surveillance systems in the world. In this guidance report "any patient with acute respiratory illness (fever and at least one sign/symptom of respiratory disease) and a history of travel to or residence in a location reporting community transmission of COVID-19 disease during the 14 days prior to symptom onset"; or "a patient with any acute respiratory illness and having been in contact with a confirmed or probable COVID-19 case" or "a patient with severe acute respiratory illness (fever and at least one sign/symptom of respiratory disease, and requiring hospitalization) and in the absence of an alternative diagnosis that fully explains the clinical presentation" has been defined as suspected cases of COVID-19; and "a suspect case for whom testing could not be performed for any reason" has been defined as a probable case (3).

Based on data collected by the WHO from national authorities (NO:185) 15,012,731 laboratory confirmed cases and 619,150 deaths related to COVID-19 have been reported till 23 July 2020 (4). Although real-time reverse transcription polymerase chain reaction (RT-PCR) has been represented as the gold standard test for defining confirmed cases, the critical role of chest CT in early and rapid diagnosis of COVID-19 cannot be denied (5, 6); some evidence has even stated that its diagnostic accuracy is equal or higher than RT-PCR (7-10). During the first few weeks of the first wave of coronavirus outbreak in Iran (February and March 2020), in some parts of the country, it was not possible to have a rapid examination of suspected cases of the disease through RT-PCR, and a large proportion of these patients have been considered as COVID-19 cases through chest CT findings. The objective of this study was to determine the most important chest CT characteristics in patients with clinical manifestations of COVID-19 who have been assessed with low-dose high resolution CT scans. 

## Methods

This observational research has been conducted as a cross-sectional study on all non-hospitalized suspected COVID-19 patients who referred to the state radiologic clinic in Babol, northern Iran to perform chest CT from March 8, 2020 to March 28, 2020. This research can be considered as a preliminary study to describe chest CT findings in patients suspected of COVID-19 infection whose result of RT-PCR examination of respiratory secretions has not yet been provided, during the first weeks of COVID-19 outbreak in Babol, northern Iran. All CT scans were reviewed by a faculty member radiologist with approximately 20 years of experience. Gender, age and service date has been recorded. Because of corona virus epidemic situation, a disposable bed sheet and surface disinfectants were used before each examination. In addition, due to respiratory symptoms in patients, especially dyspnea, scans were performed from lower parts of chest to upper parts. Computed topography was conducted using a sixteen slice multidetector (Siemens, Model: go. Now, 2019, Germany) machine. Slice thickness was set continuously on 1 millimeter. For each patient, the chest CT scan was examined for the following characteristics: 1- ground glass opacity, 2- presence of consolidative opacities, 3- unilateral or bilateral lung involvement, 4- pulmonary lobes involved, 5- degree of lung involvement (less than 20% or ≥20%), 6- other findings including round opacity, linear opacity, crazy-paving, reversed halo sign, reticular or nodular opacity, tree in bud, peribronchovascular distribution, centrilobular distribution, cavitary lesions, calcification of the aortic wall or coronary arteries, lymphadenopathy, and pleural or pericardial effusion. Imaging features have been classified into three categories: 1- highly suggestive of COVID-19 infection including images in which one of these findings has been discovered: ground glass/consolidative opacities, bilateral/multilobar involvement, peripheral distribution, round opacities, linear opacities, crazy-paving, or reversed halo sign; 2- inconsistent with COVID-19 infection including tree-in-bud opacities, centrilobular distribution, peribronchovascular distribution, predominantly nodular opacities, cavitation, lymphadenopathy, and pleural effusion (11); and 3- normal chest CTs. The sensitivity, specificity, positive predictive value (PPV), negative predictive value (NPV), positive likelihood ratio (LR+) and negative likelihood ratio (LR-) of each CT finding for diagnosing COVID-19 were calculated and reported with 95% confidence interval (95% CI). 

The collected data were analyzed with Excel 2010 and SPSS-16 software. Chi-square and logistic regression were used for data analysis. This study has been approved by the Ethics Committee of Babol University of Medical Sciences with reference code IR.MUBABOL.HRI.REC.1399.077. 

## Results

A total of 4,223 suspected patients with a range of age 9-96 years, including 2,207 (52.3%) men and 2016 (47.7%) women have been examined during three weeks. Mean age of patients was 45.51±14.72 years. Imaging characteristics in 2292 (54.3%) individuals illustrated a highly suggestive sign of COVID-19 infection; 1869 cases (44.3%) had a normal chest CT scan and 62 cases (1.4%) were inconsistent with COVID-19 infection. 

Out of 2354 individuals (55.74%) who had pulmonary involvement 1813 cases (77.00%) had bilateral and 541 cases (23.00%) had unilateral involvement; Also, 1727 (73.36%) patients had left-sided involvement. Lung field involvement in 2036 (86.49%) patients was less than 20%. Involvement of the lower lobes was more common than the other lobes; upper lobes were involved in 175 (7.43%), and middle lobe in 46 (1.95%) patients. Distribution of different imaging features showed that in 2233 (94.86%) patients with lung involvement, ground glass opacity (GGO) has been observed; and consolidative opacity has been found in 1613 (68.52%) persons. Other imaging characteristics were calcification of aortic wall or coronary arteries (285; 12.10%), linear opacity (265; 11.26%), reversed halo sign (156; 6.63%); round opacity (38; 1.61%), reticular opacity (23; 0.98%), and tree in bud (12; 0.51%). Nodular opacities (8; 0.34%), crazy-paving (6; 0.25%), peribronchovascular distribution (5; 0.21%), centrilobular distribution (4; 0.17%), pleural effusion (3; 0.13%) and lymphadenopathy (1; 0.04%) were found to have lower rates. We found no patient with cavitation in lungs or pericardial effusion. Lung opacities had predominantly a peripheral distribution. 

Association of different chest CT characteristics in highly suggestive or inconsistent COVID-19 presentation (Table 1) showed significant differences (p<0.0001). Among patients who were categorized as highly-suggestive for COVID-19 infection, 533 (26.2%) were in age-group of 50-60; 459 (22.5%) 40-50; 441 (21.6%) 30-40; 291 (14.3%) 60-70; 156 (7.7%) 20-30; 110 (5.4%) 70-80; 33 (1.6%) over 80, and 14 persons (0.7%) were less than 20 year (p<0.0001). A significant difference was found between men and women who had imaging features suggesting COVID-19 infection (p=0.002); 54.5% of them were male and 45.5% were female. The overall sensitivity, specificity, PPV, NPV, LR +and LR– of different chest CT characteristics in categorization of suspected patients as COVID-19 showed that ground glass opacity and consolidations had the highest sensitivity and specificity (Table 2); round opacities, linear opacities, crazy-paving and reversed halo sign had a sensitivity of 54% (95% CI: 53-56%). 

**Table 1:** Chest CT findings in non-hospitalized patients inconsistent or highly suggestive for COVID-19 infection

**Chest CT findings**	**Highly suggestive for COVID-19 infection** **n (%)**	**Inconsistent** ** with COVID-19 infection** **n (%)**	**p-value**
Ground glass opacity (n=2236)	2203 (98.5)	33 (1.5)	<0.001
Consolidation (n=1613)	1598 (99.1)	15 (0.9)	<0.001
calcification of aortic wall or coronary arteries (n=285)	278 (97.5)	7 (2.5)	<0.001
Linear opacity (n=266)	258 (97.0)	8 (3.0)	<0.001
Reversed halo sign (n=156)	156 (100)	0	<0.001
Round opacity (n=38)	37 (97.4)	1 (2.6)	<0.001
Reticular opacity (n=24)	2 (8.3)	22 (91.7)	<0.001
Tree in bud (n=13)	1 (7.7)	12 (92.3)	<0.001
Nodular opacities (n=8)	0	8 (100)	<0.001
Crazy-paving (n=6)	4 (66.7)	2 (33.3)	<0.001
Peribronchovascular distribution (n=5)	0	5 (100)	<0.001
Centrilobular distribution (n=4)	0	4 (100)	<0.001
Pleural effusion (n=3)	0	3 (100)	<0.001
Lymphadenopathy (n=1)	0	1 (100)	<0.001

**Table 2:** Sensitivity, specificity, PPV, NPV, LR + and LR – of ground glass opacity and consolidation features of chest CT for categorization of suspected patients as COVID-19

**Chest CT characteristics**	**Sensitivity ** **Percent (95% CI)**	**Specificity ** **Percent (95% CI)**	**PPV** **Percent (95% CI) **	**NPV ** **Percent (95% CI)**	**LR + ** ** (95% CI)**	**LR-** **(95% CI)**	**p-value**
Ground glass opacity	99 (98-99)	96 (95-96)	96 (95-97)	98 (98-99)	22 (17.96-26.9)	0.01 (0.01-0.02)	<0.001
Consolidation	99 (99-100)	73 (72-75)	70 (68-72)	99 (99-100)	3.72 (3.49-3.96)	0.01 (0.01-0.02)	<0.001

CI=confidence interval; PPV=positive predictive value; NPV=negative predictive value; LR+=positive likelihood ratio; LR-=negative likelihood ratio

## Discussion

In the first three weeks of the coronavirus epidemic outbreak in this city, an average of 201 non-hospitalized people a day have been referred to the radiologic center to be assessed for pulmonary involvement. The result is representative for high rate of reference to the state radiologic centers in order to conduct chest CTs, especially in initial and peak periods of epidemic (12). 

In this study 54.3% of non-hospitalized patients had imaging features indicating COVID-19 infection. Although chest CT is frequently used for examining respiratory involvement in suspected COVID-19 patients, its role as a screening and diagnostic tool has not been clarified. Some evidence reported that CT findings have a high sensitivity rate to detect COVID-19 infection; however, its low rate of specificity to differentiate imaging characteristics caused by different types of pneumonia, cannot be denied (12, 13). Furthermore, the diagnostic accuracy of chest CT in China has been reported to be to some extent different from other countries (12). 

Nearly half of the patients with highly-suggestive coronavirus manifestations in chest CT were in 40-60 years age-group. A systematic review and meta-analysis including 3600 individuals, found a median age of 41 years among COVID-19 patients (2). Biological differences between middle-aged, young and older adults might have significant impacts on severity and outcomes of COVID-19 infection in various age-groups. Of course, higher rate of severe disease and hospitalization is expected to be observed among elderly patients (14). 

The most common imaging features in highly-suggested cases for COVID-19 infection were ground glass opacity, with or without pulmonary consolidation. This result is in line with some previous studies. A systematic review and meta-analysis revealed that ground glass opacities might be found in 16.7-100%; consolidation as 1.6-71.5%; and GGO plus consolidation might be notified in 19.1-76.8% of patients with COVID-19 infection (13). A review article on chest CT manifestations of COVID-19 infection represented that 71.7% of patients had ground glass opacities, 41.2% consolidation, and 46.6% had both of these features (15); and another systematic review reported GGO and consolidative opacities to be as 49–94% and 11–73%, respectively (12). Radiologists with a history of 20 years or more radiologic experience claim that before the recent coronavirus epidemic began, the number of CT scans with GGO manifestation was very limited, and the observation of this imaging feature could be an important diagnostic sign (16-20). This point shows that radiologists, especially in the regions where RT-PCR examination of suspected patients is not available should be quickly and easily trained more about the CT imaging findings of COVID-19 pneumonia. 

Lower lung distribution of discovered lesions, bilateral involvement of lungs, and predominance of left lung involvement over the right lung were more evident in our study. Similar results have been reported in previous studies (15, 17, 19). Pathophysiological basis of respiratory involvement in COVID-19 disease revealed the association between the diameter of this virus and the size of the alveolar pores. When the coronavirus 2019 enters the respiratory tract, it invades the bronchioles, especially the interstitium around bronchioles at the end of lobular bronchioles, causing bronchiolitis and peribronchitis, and spreads to the distal end (20). This basis can justify the distribution pattern of pulmonary lesions. 

In our study ground glass opacity had a sensitivity, specificity, PPV, NPV, LR+ and LR- as 99%, 96%, 96%, 98%, 22 and 0.01, respectively for categorization of a patient as COVID-19 case. These values were 99%, 73%, 70%, 99%, 3.72% and 0.01%, respectively for consolidations in chest CT. A recent meta-analysis reported the pooled sensitivity, specificity, PPV and NPV of chest CT as 94% (95% CI: 91-96%), 37% (95% CI: 26-50%), 1.5-30.7%, and 95.4-99.8%, respectively to diagnose COVID-19 in symptomatic patients (21). Another research on 103 suspected COVID-19 patients in China reported these measures as 93% (85-97%), 53% (27-77%), 92% (83-96%) and 42% (18-70%), respectively (22). A prospective study in Netherlands reported a sensitivity of 89.2% (80.4–94.9%), specificity of 68.2% (58.6–76.7%), the PPV of 67.9% (61.4–73.7%), NPV 89.3% (81.6–94.0%), LR+ as 2.81 and LR- as 0.16 of the chest CT for detecting COVID-19 (23). We did not have the result of RT-PCR test to compare the diagnostic accuracy of CT findings with the gold standard measure of COVID-19 diagnosis. 

Although 44.3% of our patients had a normal chest CT, it should be emphasized that normal chest CT do not rule out a COVID-19 infection, as in some patients the targeted organ of the infection may not be the lung (24). In addition, the disease progression can determine chest CT manifestations. In the early stages of the disease, chest CT may be normal. When the disease progresses, the lesions gradually progress to multiple GGOs in the lungs, and some patients may have dense consolidations in the lung lobes (25). Although important global guidelines still introduce RT-PCR method as the gold standard for the definite diagnosis, it seems that chest CT can help medical practitioners to make COVID-19 detection in symptomatic persons and inform patients and their families more about their medical condition. (3). The strength points of this study can be represented as the large number of people whose chest CT was performed in a short time, during the first peak period of COVID-19 epidemic. Assessment of all images was done by a single skilled radiologist and special characteristics of the scanning device were used. 

We did not evaluate the nasopharyngeal samples of patients through RT-PCR to recognize the definitive cases of the disease and to compare the results with PCR tests; also, the high number of clients in the radiology clinic made it impossible to conduct a long-time interview with patients for a detailed evaluation of the disease manifestations, and these two points can be presented as the most important limitations of the present study. Since the national identification code and the contact number of the patients have been obtained, their follow-ups have been planned for future studies. 

In conclusion fifty-four percent of non-hospitalized patients suspected of COVID-19 infection who underwent a chest CT had imaging features indicating COVID-19 infection. Lower lung distribution of lesions, bilateral involvement of lungs, and predominance of the left lung were more common in people who had pulmonary involvement. Nearly 95% of patients with lung involvement showed ground glass opacities in chest CT. 
